# Metalearning: a survey of trends and technologies

**DOI:** 10.1007/s10462-013-9406-y

**Published:** 2013-07-20

**Authors:** Christiane Lemke, Marcin Budka, Bogdan Gabrys

**Affiliations:** 1 Bournemouth University, Poole House, Talbot Campus, Fern Barrow, BH12 5BB Poole, UK; 2 Unister GmbH, Leipzig, Germany

**Keywords:** Metalearning, Metaknowledge extraction, Life-long learning

## Abstract

Metalearning attracted considerable interest in the machine learning community in the last years. Yet, some disagreement remains on what does or what does not constitute a metalearning problem and in which contexts the term is used in. This survey aims at giving an all-encompassing overview of the research directions pursued under the umbrella of metalearning, reconciling different definitions given in scientific literature, listing the choices involved when designing a metalearning system and identifying some of the future research challenges in this domain.

## Introduction

The term *metalearning* first occurred in the area of educational psychology. One of the most cited researchers in this field, John Biggs, described metalearning as *being aware of and taking control of one’s own learning* (Biggs 1985). Hence, metalearning is viewed as an understanding and adaptation of learning itself on a higher level than merely acquiring subject knowledge. In that way, a person aware and capable of metalearning is able to assess his or her learning approach and adjust it according to the requirements of a specific task.

Metalearning as used in a machine learning context has many similarities to this description. Subject knowledge translates into base-learning, where experience is accumulated for one specific learning task. Metalearning starts at a higher level and is concerned with accumulating experience over several applications of a learning system according to Brazdil et al. (2009).

In the last 20 years, machine learning research was faced with an increasing number of available algorithms including a multitude of parametrisation, preprocessing and postprocessing approaches as well as a substantially extended range of applications due to increasing computing power and wider availability of computer-readable data sets. By promoting a better understanding of machine learning itself, metalearning can provide an invaluable help avoiding extensive trial and error procedures for algorithm selection, and brute force searches for suitable parametrisation. Looking at how to profit from past experience of a predictive model on certain tasks can enhance the performance of a learning algorithm and allow to better understand what makes a given algorithm perform well on a given problem.

The idea of metalearning is not new, one of the first and seminal contributions having been provided by Rice (1976). However, the literal term only started appearing in machine learning literature in the 1990s, yet still many publications deal with problems related to metalearning without using the actual word. This contribution tries to grasp every point of view metalearning has been investigated from, citing books, research and review papers of the last decade. We hope this survey will provide a useful resource for the data mining and machine learning community.

The remainder of this paper is organized as follows. In Sect. 2 we review definitions of metalearning given in scientific literature, focusing on common themes occurring in all of them. Section 3 describes different notions of metalearning, linking them to the definitions given in Sect. 2. In Sect. 4 practical considerations arising when designing a metalearning system are discussed, while open research directions are listed in Sect. 5.

## Definition

In the 1990s, the term metalearning started to appear in machine learning research, although the concept itself dates back to the mid-1970s (Rice 1976). A number of definitions of metalearning have been given, the following list cites the main review papers and books from the last decade:

1. Metalearning studies how learning systems can increase in efficiency through experience; the goal is to understand how learning itself can become flexible according to the domain or task under study (Vilalta and Drissi 2002a).
2. The primary goal of metalearning is the understanding of the interaction between the mechanism of learning and the concrete contexts in which that mechanism is applicable (Giraud-Carrier 2008).
3. Metalearning is the study of principled methods that exploit metaknowledge to obtain efficient models and solutions by adapting machine learning and data mining processes (Brazdil et al. 2009).
4. Metalearning monitors the automatic learning process itself, in the context of the learning problems it encounters, and tries to adapt its behaviour to perform better (Vanschoren 2010).

Learning systems that adapt and improve by experience are a key concept of definitions 1, 3 and 4. This in itself however does not suffice as a description, as it basically applies to all machine learning algorithms. Metalearning becomes metalearning by looking at different problems, domains, tasks or contexts or simply past experience. This aspect is inherent in all of the definitions, although somewhat disguised in definition 3 using the term *metaknowledge* instead. Metaknowledge as described by the authors stands for knowledge to be exploited from past learning tasks, which may both mean past learning tasks on the same data or using data of another problem domain. Definition 2 differs in emphasising a better comprehension of the interaction between domains and learning mechanisms, which does not necessarily imply the goal of improved learning systems, but the pursuit of a better understanding of for which tasks individual learners succeed or fail.

Rephrasing, the common ground the above definitions share, we propose to define a metalearning system as follows:

### **Definition 1**

1. A metalearning system must include a learning subsystem, which adapts with experience.
2. Experience is gained by exploiting metaknowledge extracted
  - ...in a previous learning episode on a single dataset, and/or
  - ...from different domains or problems.

Furthermore, a concept often used in metalearning is that of a bias, which, in this context, refers to a set of assumptions influencing the choice of hypotheses for explaining the data. Brazdil et al. (2009) distinguishes *declarative bias* specifying the representation of the space of hypotheses (for example representing hypotheses using neural networks only) and *procedural bias*, which affects the ordering of the hypotheses (for example preferring hypothesis with smaller runtime). The bias in base-learning according to this theory is fixed, whereas metalearning tries to choose the right bias dynamically.

## Notions of metalearning

**Fig. 1:**
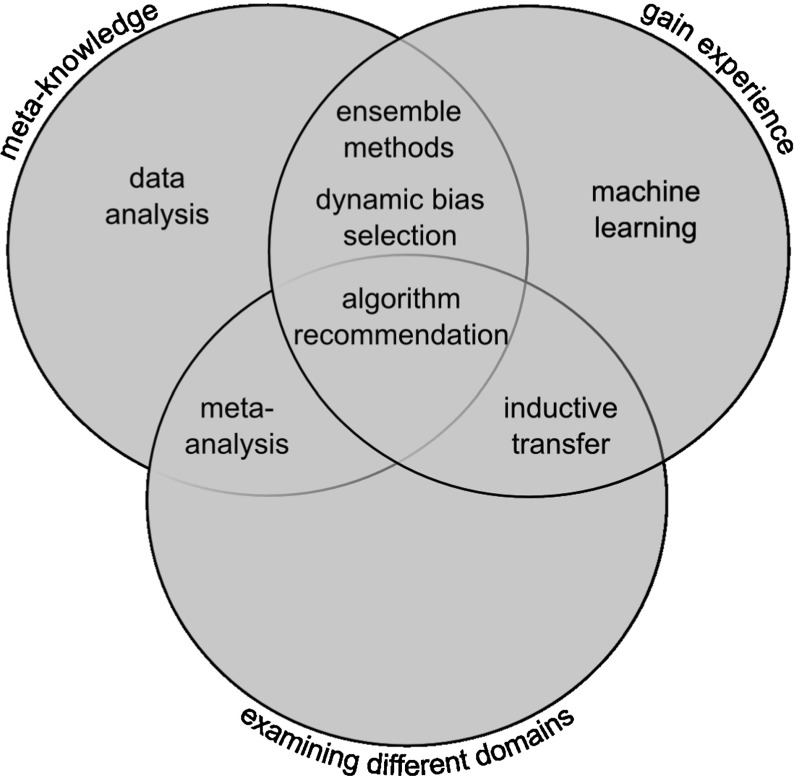
Notions of metalearning versus components of a metalearning system

Metalearning can be employed in a variety of settings, with a certain disagreement in literature about what exactly constitutes a metalearning problem. Different notions will be presented in this section while keeping an eye on the question if they can be called metalearning approaches according to Definition 1. Figure 1 groups general machine and metalearning approaches in relation to Definition 1. Each of the three circles presents a cornerstone of the definition (1: adapt with experience, 2a: meta-knowledge on same data set, 2b: meta-knowledge from different domains), the approaches are arranged into the circles and their overlapping sections depending on which parts of the definition applies to them. As an example, ensemble methods do generally work with experience gained with the same data set (definition 2a) and adapt with experience (definition 1), however, the only approach potentially applying all three parts of the definition is algorithm selection, which appears where all three circles overlap.

### Ensemble methods and combinations of base-learners

Model combination is often used when several applicable algorithms for a problem are available. Instead of selecting a single algorithm for a problem, the risk of choosing the wrong one can be reduced by combining all or a subset of the available outcomes. In machine learning, advanced model combination can be facilitated by ensemble learning according to Dietterich (2000) and Yao and Islam (2008), which comprises strategies for training and combining outputs of a number of machine learning algorithms. One often used approach of this type is resampling, leading to a number of ensemble generation techniques. Two very popular resampling-based ensemble building methods are:

- *Bagging* introduced in Breiman (1996), which denotes repeated random sampling with replacement to produce a dataset of the same size as the original training set. The dataset is subsequently used for training of a base model and the collection of models obtained in this way forms an ensemble with individual models’ decisions combined typically using voting (in classification problems) or averaging (in regression problems).
- *Boosting* proposed in Freund and Schapire (1997), which manipulates the probability with which samples are drawn from the original training data, to sequentially train classifiers focusing on the ‘difficult’ parts of the training set. Hence each consecutive ensemble member focuses on the training examples that cannot be successfully handled by the ensemble developed up to that point. The ensemble is usually built until a specified number of ensemble members is generated (although other stopping criteria are possible) and their decisions are combined using a weighted voting mechanism. Although the ensemble members can be ‘weak’ learners (i.e. models only slightly better than chance), this property must hold in the context of an increasingly difficult resampled dataset. As a result at some stage the ‘weak’ learner may in fact need to be quite complex and powerful.

The above approaches exploit variation in the data and are referred to as metalearning methods in Brazdil et al. (2009) and Vanschoren (2010). Bagging however does not satisfy point 2 of Definition 1, as consecutive random samples from the original dataset are independent from each other, so there is no experience from previous learning episodes involved. In the case of boosting however, the ensemble is built sequentially and it is the performance of previous ensemble members (i.e. experience gained while trying to solve the problem) that influences the sampling process.

More often, the following two approaches are considered as metalearning techniques:

- *Stacked generalisation* (or stacking) as introduced in Wolpert (1992), where a number of base learners is trained on the same dataset. Their outputs are subsequently being used for a higher level learning problem, building a model linking the outcomes of the base learners to the target value. The meta-model then produces the final target outcome.
- *Cascade generalisation* (Gama and Brazdil 2000), which works sequentially. When building a model, the output of the first base learner is appended to the original feature set and passed on to the next learner with the original target values. This process can then be repeated.

Although in these cases the information about base-learning is drawn in the sense of point 2a of Definition 1, these algorithms are limited to a single problem domain with a bias that is fixed a priori, so that they, using the definition above, do not undoubtedly qualify as metalearning methods.

### Algorithm recommendation

A considerable amount of metalearning research has been devoted to the area of algorithm recommendation. In this special case of metalearning, the aspect of interest is the relationship between data characteristics^1^ and algorithm performance, with the final goal of predicting an algorithm or a set of algorithms suitable for a specific problem under study. As a motivation, the fact that it is infeasible to examine all possible alternatives of algorithms in a trial and error procedure is often given along with the experts necessary if pre-selection of algorithms is to take place. This application of metalearning can thus be both useful for providing a recommendation to an end-user or automatically selecting or weighting algorithms that are most promising.

Vanschoren (2010) points out another aspect: it is not only the algorithms themselves, but different parameter settings that will naturally let performance of the same algorithm vary on different datasets. It would be possible to regard versions of the same algorithm with different parameter settings as different learning algorithms altogether, but the author advocates treating the subject and studying its effects differently. Such an approach has for example been taken in Gomes et al. (2012) and Miranda et al. (2012), where the authors discuss a hybrid metalearning and search based technique to facilitate the choice of optimal parameter values of a Support Vector Machine (SVM). In this approach, the candidate parameter settings recommended by a metalearning algorithm are used a starting point for further optimization using Tabu Search or Particle Swarm Optimization techniques, with great success. Reif et al. (2012b) investigate increasing the accuracy and decreasing runtime of a genetic algorithm for selecting learning parameters for a Support Vector Machine and a Random Forests classifier. Based on past experience on other datasets and corresponding dataset characteristics, metalearning is used to select a promising initial population for the genetic algorithm, reducing the number of iterations needed to find accurate solutions.

An interesting treatment of the above problem can also be found in Jankowski and Grabczewski (2009), where the authors propose to take into account not only the expected performance of the algorithm but also its estimated training time. In this way the algorithms can be ordered according to the estimated training complexity, which allows to produce relatively well-performing models very quickly and then look for better solutions, while the ones already trained are producing predictions. These ideas are further extended in Jankowski (2011), where some modifications of the complexity measures used are introduced.

The classic application area of algorithm selection in machine learning is classification. Smith-Miles (2008) however tries to generalise the concepts to other areas including regression, sorting, constraint satisfaction and optimisation. Metalearning for algorithm selection has also been investigated in the area of time series forecasting, where the term was first used in Prudencio and Ludermir (2004b). A comprehensive and recent treatment of the subject can be found in Wang et al. (2009) and Lemke and Gabrys (2010), where time series are clustered according to their characteristics and recommendation rules or combination weights derived with machine learning algorithms. In the area of data mining, algorithm recommendation was identified as an important research issue at the 2001 KDD conference and the 2006 KDD workshops according to Brazdil et al. (2009).

Several systems for algorithm recommendation have been implemented. Following a previous successful European commission funded project, a project named ‘meta-learning assistant for providing user support in machine learning and data mining’ (METAL 2002), investigated model selection and combination approaches. A tool resulting from this project is the data mining advisor (DMA), a web-based system providing rankings of classification algorithms for users as described in Giraud-Carrier (2005), which however is no longer accessible. The last updates on the METAL project webpage date back to 2004/2005, and the webpage is currently offline. There is however another project focussing on preprocessing for data mining with a metalearning approach, called MiningMart (Morik and Scholz 2004). Although the software tool is still available for download,^2^ its most recent version is dated October 2006.

### Dynamic bias selection

In classic algorithm recommendation, the bias depends on the available learning algorithm chosen and is not modified dynamically. Dynamic bias selection is often mentioned in relation to a continuous flow of training examples (data streams), where metalearning algorithms can be used for bias management and detecting concept drifts, as argued in Brazdil et al. (2009). In their metalearning review, Vilalta and Drissi (2002a) cite Bruha and Famili (2000) as the only example of dynamic bias selection apart from a few projects before 1992. In this contribution, a rule-based system which includes quality information about each rule, influencing the prediction process is used. The quality of a rule is updated continuously, which makes selection of the bias dynamic.

Very fast decision trees (VFDT) according to Domingos and Hulten (2000) dynamically adjust their biases with new incoming examples, with bias in this case referring to splitting tests in the tree nodes.

Another work qualifying as dynamic bias selection is the Learnt Topology Gating Artificial Neural Networks (LTGANN) by Kadlec and Gabrys (2008). In this contribution, neural networks are used for three different purposes: as base learners, as ‘local experts’ predicting the performance of one assigned base learner and as gating networks. The gating networks are used to discover relationships between different network topologies and their performance, influencing the topology of new networks added to the system.

### Inductive transfer

A different flavour of metalearning runs under the name of ‘inductive transfer’, or ‘learning to learn’. The emphasis here is not on investigating a preferably extensive set of problems as in algorithm selection; it is mostly used for a smaller selection of multiple related learning tasks. However, in the spirit of metalearning, knowledge from one problem domain is transferred across other domains or tasks. A learning mechanism thus accumulates experience that is supposed to improve performance through time. This notion puts a bigger focus on aspect 2 of Definition 1. Although aspect 1 still applies, most research in inductive transfer looks at a single learning mechanism and not a whole learning system.

Brazdil et al. (2009) distinguish two main forms of knowledge transfer using the example of neural networks. One is representational transfer, where training of the base learner is carried out sequentially. Functional transfer refers to training several neural networks in parallel while sharing all or a part of their internal structure. A special case of functional transfer is multitask learning, where a neural network uses output nodes to accommodate for more than one task. Evgeniou et al. (2005) discusses inductive transfer in kernel methods using the example of Support Vector Machines and regularisation networks. Single-task kernel learning algorithms are extended to multi-task learning by forcing all hypotheses to share a common component along with the problem-specific deviations. Kernel learning from a different perspective is presented in Aiolli (2012): a suitable kernel matrix for a linear Hebbian classifier is learnt with improving a basic kernel by learning chains of kernel transforms. Based on the results on an initial problem set, the kernel can then be applied to related problems.

In a special issue on metalearning, Silver and Bennett (2008) present several contributions in the field of inductive transfer. Zhang et al. (2008) approaches the subject from a Bayesian perspective, using a framework including latent variables for modelling a shared structure among different learning scenarios. Silver et al. (2008) uses a neural network with extra contextual inputs for different tasks. The task hence shifts to finding a single learner for the domains, only distinguished (‘indexed’) by the additional inputs.

In a recent survey article Pan and Yang (2010), inductive transfer is presented as a special case of a wider category of ‘transfer learning’, alongside transductive and unsupervised transfer learning. Transfer learning has been defined as a mechanism ‘which aims to help improve the learning of the target predictive function in a new domain, using the knowledge gained in other domains’. Although this does not strictly adhere to Definition 1, the authors discuss relevant research issues of (1) what knowledge to transfer, (2) how to transfer it, and (3) in which situations to transfer the knowledge. This last point seems especially interesting as it can be equally well cast as in which situations not to transfer the knowledge, since it may happen that the source and destination domains are not related. In such a case the performance of the new model rather than benefiting, could suffer from the knowledge transfer (so called ‘negative transfer’) (Pan and Yang 2010).

### Metalearning systems

The major usual steps of a modelling process consist of data analysis, data preprocessing, model building and a phase of interpretation and evaluation. Rather than applying metalearning in only a part of this process, latest research started taking a more holistic view by investigating metalearning frameworks and architectures.

While not yet mentioned in the seminal review of Vilalta and Drissi (2002a), Brazdil et al. (2009) give an overview of (semi-)automatic metalearning systems for data mining, only two of which target more than one aspect of the knowledge discovery process: Project CITRUS according to Wirth et al. (1997) seems to have been discontinued after a few publications in 1996 and 1997, however, it did target the complete modelling process. A bit more recently, Bernstein et al. (2005) propose the Intelligent Discovery Assistant (IDA), providing a template for ontology-driven assistants for knowledge discovery dealing with preprocessing, model building and post-processing. A realization of this idea has been materialized within the e-LICO project,^3^ which investigated the concept of ‘meta-mining’—ontology and metaknowledge driven recommendation of data mining workflows as proposed in Nguyen et al. (2011) and evaluated in Nguyen et al. (2012). A somewhat related term, ‘domain-driven data mining’, also gained some recognition in the last years. The idea promotes a ubiquitous intelligence to be incorporated into the data mining process from a more methodological point of view, increasing reusability and understanding of algorithms. An overview of research done in this area and a discussion of future challenges and issues can be found in Cao (2010).

For continuous streams of data and the example of softsensors in process industry, Kadlec and Gabrys (2009) develop an architecture promoting life-long learning, where base-learners and pre- and post-processing methods can be plugged in and dynamically combined. A metalearning component optimises the system with regard to a global performance function. Jankowski and Grabczewski (2009) describe the implementation of an extensive metalearning architecture in detail, dealing with aspects like algorithm selection and parameter tuning, with the latter topic also addressed in Molina et al. (2012) for automatic tuning of parameters of decision trees.

METALA is an agent-based architecture with a metalearning component, which has been implemented as a J2EE based framework with details given in Hernansaez et al. (2004). It supports an arbitrary number of algorithms, which are managed by agents. Using metalearning on statistical and information-theoretic task properties, the best algorithm is dynamically selected, if the pool of algorithms and tasks is updated.

A distinct concept of lazy metalearning has been extensively studied in Bonissone (2012). The motivation for this work is the apparent lack of automation in model creation, which leads to bottlenecks in the models lifecycle and scalability (two other attempts at automation of model development methodology can also be found in Budka and Gabrys 2010; Budka et al. 2010). The main premise in lazy metalearning is that there is access to a large library of both local and global, pre-computed models together with their meta-information, which are perceived as commodity and which have been enabled by the recent expansion of cloud computing and crowdsourcing. The idea is to build dynamic on-demand ensembles using the models from the library as the building blocks, rather than optimizing and tuning of pre-computed models (Bonissone 2012). Since exhaustive enumeration of all possible combinations of models in a library would be intractable, a multi-step selection process based on metaknowledge, query information and correlation of errors of the ensemble members is employed. According to Bonissone (2012), a multi-criteria decision making process is followed in order to (1) create the model by pre-selecting the initial building blocks for the assembly and compiling their meta-information, which is an off-line phase, and (2) perform dynamic model assembly, where the best subset of models for a given query is selected on-line, i.e. during runtime. The process is also able to determine the weights of the ensemble models in the fusion schema, based on their local performance around the query. Some other recent approaches to development of metalearning frameworks can be found in Matijaš et al. (2013) for the problem of electricity load forecasting, Abbasi et al. (2012) for financial fraud detection or Tsai and Hsu (2013) for bankruptcy prediction.

## Considerations for using metalearning

Before applying metalearning to any problem, certain practical choices have to be made. This includes the choice of a metalearning algorithm, which can even constitute a meta-metalearning problem itself. Selection of appropriate metaknowledge and the problem of setting up and maintaining metadatabases have to be tackled, research efforts of which will be summarised in this section.

### Prerequisites

As also elaborated on in Brazdil et al. (2009), metalearning can not be seen as a magic cure to machine learning problems for a variety of reasons. First of all, the extracted metafeatures need to be representative of their problem domain, otherwise, an algorithm will fail to identify similar domains. On the same note, if a problem has not been seen before, metalearning will be unable to exploit past knowledge to improve prediction performance. Performance estimation may be unreliable because of the natural limitations of estimating the true performance of the dataset. Different metafeatures might be applicable to each dataset. These issues emphasise the importance of being critical when designing a metalearning system.

### Metalearning algorithms

Vanschoren (2010) gives a survey on efforts to describe properties of algorithms. The author distinguishes qualitative properties (for example type of data that can be handled, learning strategy, incrementality) and quantitative properties (bias-variance profile, runtime properties like scalability and resilience). In an effort to find an implementation and vendor-independent method for representing machine learning models, the XML-based standard PMML has been developed and gained some recognition in the last years. A detailed description of PMML can be found in Guazzelli et al. (2009).

The choice of a metalearning algorithm naturally depends on the problem and the task to be solved. Generally, traditional classification algorithms are very successful in metalearning algorithm selection and can include meta-decision trees (Todorovski and Dzeroski 2003), neural networks, Support Vector Machines or any other classification algorithms, with the *k*-Nearest Neighbours being another popular choice (Brazdil et al. 2009). Applying regression algorithms is less popular, even smaller is the number of available algorithms to learn rankings. One of the simplest ranking method involves dividing the problem space using clustering of available datasets according to a distance measure (usually *k*-Nearest Neighbour) of the metafeatures and using average performance ranks of the cluster into which a new problem falls (Brazdil et al. 2003). Brazdil and Soares (2000) also look at the magnitude and significance of the differences in performance. The NOEMON approach introduced by Kalousis and Theoharis (1999) builds classifiers for each pair of base forecasting methods with a ranking being generated using the classifiers’ outputs. Todorovski et al. (2002) build decision trees using the positions in a ranking as target values.

### Extracting metaknowledge

According to Brazdil et al. (2009), metaknowledge is derived in the course of employing a learning system. A very common form of metaknowledge is the performance of algorithms in certain problem domains, which is to be linked with characteristics of the task. Several possibilities for characterising a problem domain exist.

The most straightforward form of metaknowledge extracted from the data include statistical or information-theoretic features. For classification problems, Brazdil et al. (2009) mention the number of classes and features, ratio of examples to features, degree of correlation between features and target concept and average class entropy. For other application areas, features can look completely different, as for example summarised in Lemke et al. (2009) for the area of time series forecasting, where features can include, for example, length, seasonality, autocorrelation, standard deviation and trends of the series.

Vilalta and Drissi (2002b) propose measures for the difficulty of a classification problem that can be used as an input for metalearning. They include class variation, denoting the probability that, by means of a distance measure, any two neighbouring data records have a different class value and example cohesiveness, measuring the density of the example distribution in the training set. In a similar approach, Köpf and Iglezakis (2002) also suggest comparing observations with each other and extract ‘case base properties’, which assess the quality of a dataset using measures such as redundancy, for example induced by data records that are exactly the same, or incoherency, which, for example occurs if data records have the same features but different class labels.

Alternatively to looking at the data only, information of individual algorithms and how they solved the problem can be considered, for example their predicted confidence intervals. This can be achieved by using a model that is fast to build and train and investigating its properties. In this spirit, Bensusan et al. (2000) suggest building a decision tree for a classification problem and using properties of the tree such as nodes per feature, tree depth or shape to characterise it. Another approach is landmarking as proposed in Pfahringer et al. (2000), using the performance of simple algorithms to describe a problem and correlating this information with the performance of more advanced learning algorithms. A list of landmarking algorithms can be found in Vanschoren (2010). Landmarking algorithms can also be run on only a small sample of the data available, reducing the training time required. Performance information of different algorithms and learning curves generated when more data is added to the training set can then be used to select an algorithm according to Fürnkranz et al. (2002).

Empirical evaluation of different categories of metafeatures in the context of their suitability for predicting classification accuracies of a number of standard classifiers can be found in Reif et al. (2012c). The authors distinguish 5 such categories of features i.e. simple, statistical, information-theoretic, landmarking and model-based, which corresponds to the general categorization evident from the literature.

As with any learning problem, metalearning is subject to the ‘curse of dimensionality’ (Bishop 1995) and other issues, which can traditionally be solved by selecting a subset of relevant features. Although to the best of our knowledge, in the context of metalearning this issue has only been addressed in relatively few publications (e.g. Kalousis and Hilario 2001; Reif et al. 2012c; Todorovski et al. 2000), we assume that the reason for this is quite simple—meta-feature selection does not differ from feature selection at the base-level, and the machine learning literature is very rich in this regard (a comprehensive review of various feature selection techniques can be found in Guyon and Elisseeff 2003).

### Metadatabases

As metalearning profits from knowledge obtained while looking at data from other problem domains, having sufficient datasets at one’s disposal is important. Soares (2009) propose transforming existing datasets (‘datasetoids’) to obtain a larger number of them and show success of the approach on a metalearning post-processing problem. Vanschoren (2010) states that there is no lack of experiments being done, but datasets and information obtained often remain in ‘people’s heads and labs’. He proposes a framework to export experiments to specifically designed experiment databases based on an ontology for experimentation in machine learning. The resulting database can then, for example, give information on rankings of learning algorithms, the behaviour of ensemble methods, learning curve analyses and the bias-variance behaviour of algorithms. One example of such database can be The Open Experiment Database.^4^ An analysis of this database together with a critical review can be found in Driessens et al. (2012).

An alternative approach to the problem of scarcity metadatabases has been presented in Reif et al. (2012a), where the authors describe a dataset generator able to produce synthetic datasets with specified values of some metafeatures (like kurtosis and skewness). Although the proposed generator appears to be at a very early stage of development, the idea is definitely very promising, also from the point of view of performing controlled experiments on datasets with specified properties. Similarly to feature selection, synthetic data generation has received a considerable attention in the recent generic machine learning and data mining literature, especially in the context of data streams and concept drift (please see Bifet et al. 2011 and references therein).

## Conclusions and research challenges

Research in the area of metalearning is continuing in several directions. One area is the identification of metafeatures. As mentioned before, the vast majority of publications investigates extracting features from the dataset, mostly in the form of statistical or information theoretic measures. Landmarking is a different approach using simple base learning algorithms and their performance to describe the dataset at hand. However, Brazdil et al. (2009) argue that characteristics of learning algorithms and gaining a better understanding of their behaviour would be a valuable research avenue with very few publications, for example Vanschoren and Blockeel (2006), that exist in this area to date.

A lot of publications on metalearning focus on selecting the base-learning method that is most likely to perform well for a specific problem. Fewer publications like Brazdil et al. (2003) and Prudencio and Ludermir (2004a) consider ranking algorithms, which can be used to guide combination weights and to increase robustness of a metalearning system.

Regarding adaptivity and continuous monitoring, many approaches go further than the static traditional metalearning approaches, for example by using architectures that support life-long learning such as in Kadlec and Gabrys (2009). However, research in this area can still go a long way further investigating continuous adjustment, rebuilding or discarding of base-learners with the help of metalearning approaches.

Users of predictive systems are faced with a difficult choice of an ever increasing number of models and techniques. Metalearning can help to reduce the amount of experimentation by providing dynamic advice in form of assistants, decrease the time that has to be spent on introducing, tuning and maintaining models and help to promote machine learning outside of an academic environment. In this context, architectures and frameworks using metalearning have been named in Sect. 3.2. However, many of these are work in progress, no longer maintained or tackle only one of the aspects in a modelling process, so this area would benefit from further research and implementations as well.

## Footnotes

1 In a more advanced form it is the relationship between data and algorithm characteristics, and algorithm performance.

2
http://www-ai.cs.uni-dortmund.de/MMWEB/downloads.

3
http://www.e-lico.eu/.

4
http://expdb.cs.kuleuven.be/.
